# Numerical simulation of reservoir parameters’ synergetic time-variability on development rules

**DOI:** 10.1007/s13202-015-0208-4

**Published:** 2015-11-06

**Authors:** Jian Hou, Yanhui Zhang, Daigang Wang, Kang Zhou

**Affiliations:** 1 State Key Laboratory of Heavy Oil Processing, China University of Petroleum, Qingdao, China; 2 College of Petroleum Engineering, China University of Petroleum, Qingdao, China

**Keywords:** Water flooding, Network simulation, Reservoir numerical simulation, Reservoir parameter, Time variability

## Abstract

Time variability of reservoir parameters in water flooding has an effect on oilfield development rules. Meanwhile, time variability of different reservoir macro-parameters has certain synergetic relationship with each other. Based on microscopic network simulation and reservoir numerical simulation, a new simulation method is presented, which can describe the influence of reservoir parameters’ synergetic time-variability on oilfield development rules both in macroscopic and microscopic scales. Microscopic network simulation can effectively simulate the impact of micro-parameters’ variation on macro-parameters, thus a comprehensive model is built to reflect the variability of reservoir parameters. On the basis of considering time variability of porosity, permeability, and relative permeability in water flooding, an improved reservoir numerical simulator is established, which can effectively simulate the effect of reservoir parameters’ synergetic variation on oilfield development rules.

## Introduction

Long-term water flooding in oilfields has a strong, complex, and abiding dynamic geology effect on reservoirs, making reservoir microstructures change gradually, thus causing the variation of reservoir macro-parameters (Li 2005). The time variability of reservoir parameters must have an impact on oilfield development rules. There is some internal relationship between the variation of reservoir macro-parameters (such as porosity, permeability, and relative permeability curve) and the variation of micro-parameters (such as pore radius, aspect ratio, shape factor, and coordination number). Change of reservoir macro-parameters is substantially a reflection of reservoir micro-parameters’ variation in macroscopic scale. Therefore, time variability of different reservoir macro-parameters should have certain synergetic relationship with each other. However, studies at present are confined to describing the change law of individual reservoir parameter (Deng and Xu 2004; Jackson et al. 2005; Wang et al. 2004) with less focus on investigating the interrelation between the variation of each individual reservoir parameter.

Network modeling is a useful tool for investigating pore-scale behavior and in some cases for determining macroscopic information such as permeability (Bryant et al. 1993), relative permeability (Bakke and Øren 1997), and capillary pressure (Dillard and Blunt 2000). Several studies have successfully used network models to investigate effect of wettability variation on flow (Hui and Blunt 2000; Jackson et al. 2003; Suicmez et al. 2008; Tripathi 2009) and to investigate effect of relative permeability in reservoir simulation of water-alternating-gas injection (Kossack 2000; Spiteri and Juanes 2006; Suicmez et al. 2007). In these models, some reservoir parameters have been discussed separately; however, until recently, there has been less focus on investigating the interrelation between the variations of individual reservoir parameter.

When describing the influence of reservoir parameters’ variability on oil development rules, the simulation technique which uses a pore-scale network model in conjunction with reservoir-scale conventional simulations is needed. Recently, several studies have tried to capturing the relevant flow physics at different scales. KlØv et al. (2003) made a representative study on upscaling technique. They computed relative permeability and capillary pressure curve using network modeling techniques (μm-scale) and determined effective flow properties at the heterogeneous facies scale (m-scale). Finally, the effective flow properties are implemented in a field scale (km-scale) simulation model. Balhoff et al. (2007) coupled physically representative network models created from computer-generated sphere packings to adjacent continuum-scale models. Furthermore, Rhodes et al. (2009) proposed a pore-to-field transport simulation approach and applied it to single-phase flow accounting for advection and diffusion. In their study, particle transition from pore to pore is modeled as a continuous-time random walk (CTRW), and the reservoir is represented as a network of nodes connected by links.

Combined with microscopic network simulation and reservoir numerical simulation, a comprehensive model is built to describe the variability of reservoir macro-parameters in this paper, which can describe the internal relationship of macro-parameters, micro-parameters, and oilfield development rules. In this work, a novel approach is proposed to carry out reservoir engineering study in both macroscopic and microscopic scales, and a new research method is proposed, which can represent the influence of reservoir parameters’ synergetic time-variability on oilfield development rules.

## Microscopic simulation method

To build a comprehensive model describing the variability of reservoir macro-parameters, one needs to investigate the relationship between the variability of reservoir macro-parameters and that of micro-parameters. The network model applies modeling networks to substitute the complex porous space in porous media, and studies the flow mechanism in porous media using random simulations at the microscopic stage (Blunt et al. 2002; Hou 2007; Mahmud et al. 2007; Hou et al. 2011a, b). By adjusting micro-parameters of the network model, it can simulate the impact of reservoir micro-parameters’ variation on macro-parameters (Hou et al. 2011a, b), and thus, it provides an effective tool to describe the internal relationship between reservoir macro-parameters and reservoir micro-parameters. The network model consists of pores and throats, which represent large and narrow void spaces, respectively.

Initially, the network is fully saturated with water and is strongly water-wet. Primary drainage is used to simulate the formation of a reservoir. At the beginning of displacement, the displacing fluid (oil) is injected from the entrance. As the pressure drop between the two ends of the model gradually increases, the displacing fluid enters the network model to displace fluid until the water saturation or capillary pressure reaches a given value. The invasion percolation method (Wilkinson and Willemsen 1983) is adopted at every step during displacement: units (pore or throat) with the lowest threshold pressure are selected for the entrance of the displacing fluid. Based on the MS-P method, the threshold pressure *P*_c_ of a capillary tube for pore-body filling can be determined by

1 $$P_{\text{c}} = \frac{{\sigma_{\text{ow}} \left( {1 + 2\sqrt {\pi G} } \right)\cos \theta_{\text{r}} }}{r}F_{\text{d}} \left( {\theta_{\text{r}} ,G} \right),$$

where *σ*_ow_ is the interfacial tension, *G* is the shape factor, *r* is the inscribed radius, *θ*_r_ is the receding contact angle, and *F*_d_ is a function of *θ*_r_ and *G*.

After primary drainage, the imbibition process of water flooding is carried out. Due to the change in wettability of some pores, some water remains in the corners. The mechanism of waterflooding is much more complex than that of primary drainage. Lenormand et al. (1983) described displacement mechanisms at the pore level of water-wet and partial water-wet systems. There are three types of displacement: piston-type, pore-body filling, and snap-off displacement.

The macro-parameters such as porosity, permeability, and relative permeability are used as constraint conditions to generate the network model. A three-dimensional network model is established by selecting core data for the sake of simulation, which is represented by the typical relative permeability curve of the whole mount sandstone reservoirs with high permeability in Shengli oilfield. The absolute permeability is 2 μm^2^, and the porosity is 34.5 %.

**Fig. 1:**
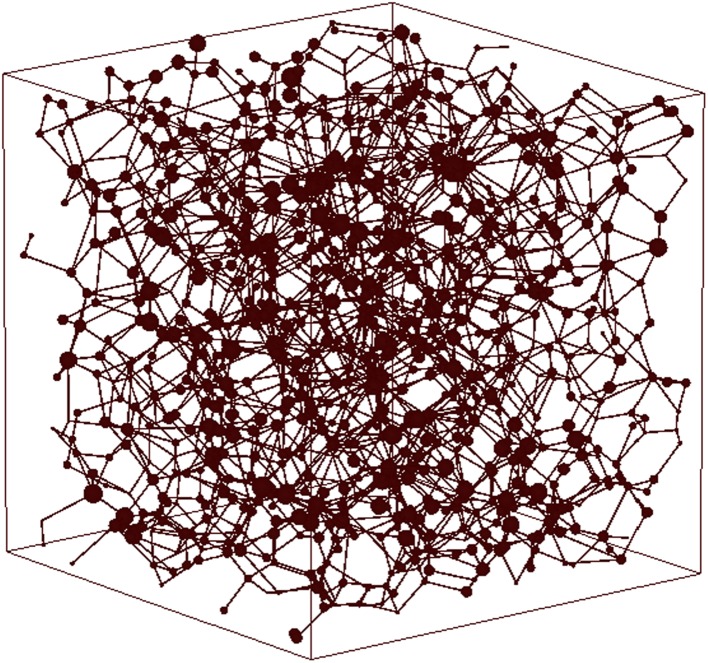
Three-dimensional network model

In process of fitting the macro-parameters, the related micro-parameters such as pore structure parameters and surface properties parameters are adjusted properly to establish the network model. As shown in Fig. 1, balls represent the pores and line segments represent throats, with 1147 pores and 2265 throats altogether. Size of balls reflects different spatial dimension of pore bodies. The pore radius varies from 14.8 to 65.4 μm. The average coordination number is 4.32; the porosity is 35.6 %; the absolute permeability is 2.129 μm^2^. Other fundamental parameters of network modeling are listed in Table 1.

**Table 1:** Fundamental parameters of network modeling

Parameter	Value	Parameter	Value
Network size	1.60 mm × 1.59 mm × 1.59 mm	Proportion of triangular pore throat	85 %
Pore throat radius	14.8–65.4 μm	Proportion of square pore throat	5 %
Throat radius	2.57–27.0 μm	Proportion of circular pore throat	10 %
Average throat radius	14.44 μm	Shape factor of triangular pore throat	0.032
Throat radius uniformity coefficient	0.54	Intrinsic contact angle	45°–63°
Throat length	0.31–199.9 μm	Average intrinsic contact angle	53°
Aspect ratio	1.1–6.5	Oil/water interfacial tension	30 mN/m
Average aspect ratio	2.328	Water density	1.0 g/cm^3^
Average coordination umber	4.32	Oil density	0.88 g/cm^3^
Porosity	35.6 %	Water viscosity	1.0 mPa s
Absolute permeability	2.129 μm^2^	Oil viscosity	16.42 mPa s
Water compressibility	4.35 × 10^−4^ MPa^−1^	Oil compressibility	1.45 × 10^−3^ MPa^−1^

**Fig. 2:**
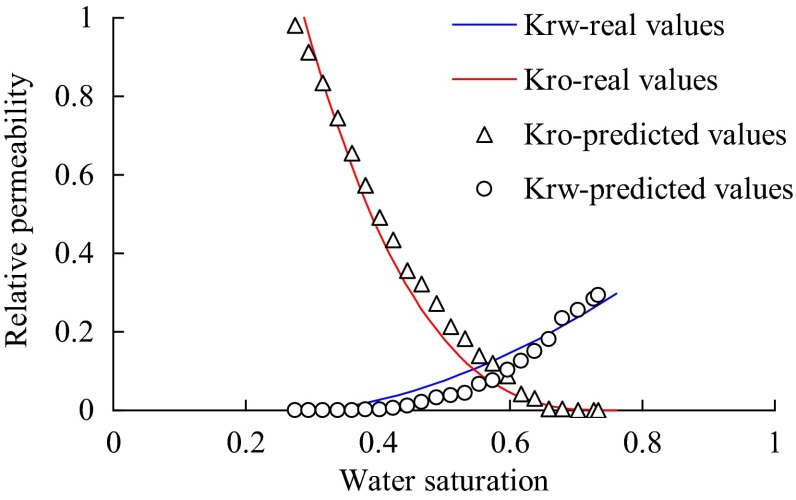
Fitting of relative permeability curve

Fitting result of relative permeability curve is shown in Fig. 2, among of which, the discrete points denote the predicted water–oil relative permeability curve by network modeling the imbibition process, and the solid lines correspond to the typical relative permeability curve of the whole mount sandstone reservoirs with high permeability in Shengli oilfield. The predicted irreducible water saturation and residual oil saturation are 28.85 and 24.01 %, respectively. According to the typical relative permeability curve, the real values of endpoint saturation are 27.47 and 26.74 %, respectively. From analysis, it can be found that not only the endpoint saturations but also the trend of curve fitting is consistent.

## Macroscopic simulation method

Prediction and simulation on the production performance of oilfields are carried out in macroscopic scale. By adjusting the reservoir macro-parameters such as porosity, permeability, and relative permeability curve, the influence of reservoir parameters’ variation on oilfield development rules can be studied, and then, the internal relationship between reservoir macro-parameters and oilfield development rules can be established.

The model of oil–water two-phase flow is based on the following basic assumptions: the seepage flow of reservoirs is carried out at a certain temperature, there are oil phase and water phase in reservoirs, each phase follows the Darcy’s law, fluids and rocks are both compressible, and the influence of capillary pressure and gravity is considered. The mass conservation equations for different components are expressed as

As for water component,

2 $${\nabla } \times \left[ {\frac{{KK_{\text{rw}} }}{{B_{\text{w}} \mu_{\text{w}} }}\left( {{\nabla }P_{\text{w}} - \gamma_{\text{w}} {\nabla }Z} \right)} \right] + q_{\text{wsc}} = \frac{\partial }{\partial t}\left( {\frac{{\phi S_{\text{w}} }}{{B_{\text{w}} }}} \right)$$

As for oil component,

3 $${\nabla } \times \left[ {\frac{{KK_{\text{ro}} }}{{B_{\text{o}} \mu_{\text{o}} }}\left( {{\nabla }P_{\text{o}} - \gamma_{\text{o}} {\nabla }Z} \right)} \right] + q_{\text{osc}} = \frac{\partial }{\partial t}\left( {\frac{{\phi S_{\text{o}} }}{{B_{\text{o}} }}} \right),$$

where *K* is the formation permeability, *ϕ* is the porosity, *K*_ro_ and *K*_rw_ are, respectively, oil-phase and water-phase relative permeability, *S*_o_ and *S*_w_ are, respectively, oil-phase and water-phase saturation, *P*_o_ and *P*_w_ are, respectively, oil-phase and water-phase pressure, *q*_osc_ and *q*_wsc_ are, respectively, oil-phase and water-phase volume injected or produced per volume of unit rock, per unit time in ground conditions, *B*_o_ and *B*_w_ are, respectively, oil-phase and water-phase volume factor, *γ*_o_ and *γ*_w_ are, respectively, oil-phase and water-phase gravity, *μ*_o_ and *μ*_w_ are, respectively, oil-phase and water-phase viscosity, *Z* is the formation depth, and *t* is the production time.

Time variability of reservoir parameters must have an effect on oilfield development rules. However, most of reservoir numerical simulators commonly used at present do not describe the variability of reservoir parameters. Injecting fluid for a long time is the basic reason of reservoir attaints for parameters’ variation. To improve the reservoir numerical simulator, a parameter related with injected water volume should be determined to establish the relationship between it and reservoir parameters’ variation.

Initially, an evaluation index (scour pore volume multiple) was introduced to quantitatively represent the erosion strength of injected water on reservoir. The scour pore volume multiple of each grid *F*_DG_ is defined as

4 $$F_{\text{DG}} = \frac{{Q_{\text{TG}} }}{{V_{\text{PG}} \times N}},$$

where *Q*_TG_ is the total flow of grid, namely the total of cumulative inflow or cumulative outflow in *X* direction, *Y* direction, and *Z* direction, *V*_PG_ is the pore volume of grid, and *N* is the total number of grids simulated. Therefore, the scour multiple of injected water *F*_DG_ is regard to be defined in a sense of the whole simulation area, which is essentially consistent with the physical meaning of the scour multiple of injected water defined in laboratory experiments.

### Model modification for permeability’s time-variability

Fluid erosion to formation has the greatest effect on permeability. A secondary variation pattern is presented considering different change speeds of reservoir parameters in water flooding. The change multiple of permeability can be determined by

5 $$R_{K} = \frac{K}{{K_{0} }} = \frac{{R_{{K{ \hbox{max} }}} }}{{F_{\text{DGmax}}^{2} }}F_{\text{DG}}^{2},$$

where *K*_0_ is the initial permeability of every grid, *R*_*K*max_ is the largest change multiple of permeability, *F*_DGmax_ is the largest scour pore volume multiple. If *F*_DG_ ≥ *F*_DGmax_, *R*_*K*_ = *R*_*K*max_. In the process of improving the reservoir numerical simulator, the conductivity needs to be calculated again after modifying the permeability field.

### Model modification for porosity’s time-variability

The change multiple of porosity is defined as

6 $$R_{\phi } = {\phi \mathord{\left/ {\vphantom {\phi {\phi_{0} }}} \right. \kern-0pt} {\phi_{0} }},$$

where *ϕ*_0_ is the initial porosity of every grid. On the basis of considering the time variability of porosity, it is also necessary to consider the influence of compressibility on porosity simultaneously.

Considering that modification of porosity will lead to variability of the original oil in place, it will influence the material balance of underground crude oil. Therefore, when modifying the porosity, the corresponding modification of oil saturation is carried out:

7 $$S_{\text{oh}} = \frac{{\phi_{q} S_{\text{oq}} }}{{\phi_{h} }},$$

where $S_{\text{oq}} ,\phi_{q}$ are, respectively, oil saturation and porosity before modification, and $S_{\text{oh}} ,\phi_{h}$ are, respectively, oil saturation and porosity after modification.

### Model modification for relative permeability’s time-variability

The method of modifying relative permeability is described as follows. Firstly, it is necessary to determine the variability of every controlling parameter of relative permeability curve (such as irreducible water saturation, residual oil saturation, and ratio of endpoint relative permeability), then the parameters’ variations are substituted into the correlative empirical formula of relative permeability to modify the relative permeability curve.

The relative permeability of oil–water two-phase flow can be determined by the following correlative empirical formula (Qin and Li 2001):

8 $$S_{\text{w}}^{*} = \frac{{S_{\text{w}} - S_{\text{wc}} }}{{1 - S_{\text{wc}} - S_{\text{or}} }}$$

9 $$K_{\text{rw}} = \frac{{K_{\text{rwro}} }}{{K_{\text{rocw}} }}\left( {S_{\text{w}}^{*} } \right)^{{n_{\text{w}} }}$$

10 $$K_{\text{ro}} = \left( {1 - S_{\text{w}}^{*} } \right)^{{n_{\text{o}} }},$$

where *S*_w_ is the water saturation, *S*_wc_ and *S*_or_ are, respectively, irreducible water saturation and residual oil saturation, *K*_rocw_ and *K*_rwro_ are, respectively, oil-phase relative permeability at irreducible water saturation and water-phase relative permeability at residual oil saturation, and *n*_o_ and *n*_w_ are, respectively, relative permeability exponents of oil-phase and water-phase.

The change value of irreducible water saturation *D*_swc_, the change value of residual oil saturation *D*_sor_, and the change multiple of ratio of endpoint relative permeability can be respectively determined by

11 $$D_{\text{swc}} = S_{\text{wc}} - S_{\text{wc0}}$$

12 $$D_{\text{sor}} = S_{\text{or}} - S_{{{\text{or}}0}}$$

13 $$R_{\text{kr}} = {{K_{\text{rwo}} } \mathord{\left/ {\vphantom {{K_{\text{rwo}} } {K_{{{\text{rwo}}0}} }}} \right. \kern-0pt} {K_{{{\text{rwo}}0}} }},$$

where *K*_rwo_ is the ratio of endpoint relative permeability, *K*_rwo_ = *K*_rwro_/*K*_rwro_, and *S*_wc0_, *S*_or0_, *K*_rwo0_ are the initial irreducible water saturation, residual oil saturation, and ratio of endpoint relative permeability, respectively.

Different from the modification of porosity and permeability on every grid, the relative permeability curve selected for simulation is modified at every time step of calculation.

## Simulation procedure

Although the change mechanisms of reservoir parameters (porosity, permeability, and relative permeability curve included) are complex, the variability of these parameters has some internal relation with each other. Based on microscopic network simulation and reservoir numerical simulation, a new simulation method is proposed, which considers the synergetic time-variability of reservoir parameters in macroscopic and microscopic scales. Procedures of simulation consist of

1. Macro-parameters such as porosity, absolute permeability, and relative permeability are used as constraint conditions to generate the microscopic network model of oil–water two-phase flow.
2. Based on network simulation, the change relationship between reservoir macro-parameters and micro-parameters is studied. A comprehensive model describing the variability of reservoir macro-parameters is proposed using a statistical analysis technique, namely the relationship of change multiple of porosity *R*_*ϕ*_, change value of irreducible water saturation *D*_swc_, change value of residual oil saturation *D*_sor_, change multiple of ratio of endpoint relative permeability *R*_*kr*_, and change multiple of permeability *R*_*k*_.
3. Establish a reservoir simulator for macroscopic numerical simulation, and input the petrophysical parameters, the initial relative permeability curve, the initial reservoir parameter field, and the production performance.
4. The relationship between the change multiple of porosity and the scour pore volume multiple is built using a secondary variation pattern.
5. The procedures that need to be accomplished at every time step of reservoir numerical simulation can be summarized as follows: (1) calculate the scour pore volume multiple of injected water; (2) calculate the change multiple of permeability *R*_*K*_ with Eq. (5); (3) calculate the average permeability change multiple of all grids. On this basis, using the comprehensive model of reservoir macro-parameters, calculate the change value of irreducible water saturation *D*_swc_, the change value of residual oil saturation *D*_sor_, the change multiple of ratio of endpoint relative permeability *R*_*kr*_, and modify the relative permeability curve as a whole; (4) modify permeability of every grid and recalculate conductivity; (5) calculate the change multiple of porosity according to the comprehensive model of reservoir macro-parameters, meanwhile, modify porosity of every grid considering the influence of compressibility on porosity, and then modify the distribution of fluid saturation considering the material balance; (6) solve the pressure and saturation equation.
6. Investigate the influence of reservoir parameters’ variation on oilfield development rules by considering the synergetic time-variability of permeability, porosity, and relative permeability curve.

## Comprehensive model for reservoir macro-parameters’ time-variability

Due to long-term erosion of injected water, reservoir micro-parameters yield some changes in water flooding, especially in high water cut stage. Concretely speaking, the variations can be summarized as follows: throat radius becomes larger; homogeneity of pore throat increases; aspect ratio decreases; pore bodies and throats become more regular; shape factor becomes bigger; coordination number increases; and wettability varies toward water-wet. All the micro-parameters’ variations have an integrated effect on reservoir macro-parameters, namely, result in the variability of porosity, permeability, and relative permeability. For instance, porosity and permeability increase; irreducible water saturation becomes larger; residual oil saturation decreases; and ratio of endpoint relative permeability augments (Sun 2002).

To establish the comprehensive model for reservoir macro-parameters time-variability, the impact of different reservoir micro-parameters’ variation on macro-parameters such as porosity, permeability, and relative permeability should be studied primarily using network modeling (Hou et al. 2011a, b). The micro-parameters mainly include throat radius, throat radius uniformity coefficient, aspect ratio, wettability, shape of pore throat, and coordination number.

To compare the influence degree of different micro-parameters on reservoir macro-parameters and determine the main influential factors, the variance coefficient is selected as the evaluation index, which takes the form of Eqs. 14 and 15.

14 $$C_{\text{v}} = \frac{S}{{\left| {\bar{y}} \right|}}$$

15 $$S = \sqrt {\frac{1}{n - 1}\sum\limits_{i = 1}^{n} {(y_{i} - \bar{y})^{2} } }$$

where *C*_v_ is the variance coefficient, *S* is the standard deviation of data sample, $\left| {\overline{y} } \right|$ is the absolute mean value, *n* is the number of data points, and *y*_*i*_ is the *i*th data point. Variance coefficient *C*_v_ can reflect the difference degree of data points. The bigger the *C*_v_, the larger the difference degree of data points, or else, the data sample will be more concentrative. Considering that value of the variance coefficient can represent the sensitivity of various factors on reservoir macro-parameters, we can then determine the main influential factors of reservoir macro-parameters.

The variance coefficients of different reservoir micro-parameters’ influence degree are concluded in Table 2. If we sort the data points of different columns in Table 2 from maximum to minimum, the major and minor influential factors of various reservoir macro-parameters can be obtained. The influential factors whose variance coefficient is greater than the mean variance coefficient of the same column are picked out and marked by the symbol “*”, which denote that the micro-parameters labeled specially are the main influential factors of the reservoir macro-parameter.

**Table 2:** Variance coefficient of different influential factors’ influence degree

Influential factors	*C*_v_(*ϕ*)	*C*_v_(*K*)	*C*_v_(*S*_wc_)	*C*_v_(*S*_or_)	*C*_v_(*K*_rwo_)
Throat radius	0.167*	0.469*	0.008	0.084	0.078
Throat radius uniformity coefficient	0.005	0.291*	0.006	0.105	0.290*
Aspect ratio	0.005	0.324*	0.006	0.492*	0.442*
Wettability	0.000	0.000	0.181*	0.090	0.079
Shape of pore throat	0.006	0.077	0.259*	0.215*	0.116
Coordination number	0.003	0.303*	0.023	0.180*	0.291*
Mean	0.031	0.244	0.081	0.194	0.216

On the basis of analysis, it can be found that the main influential factor of porosity is throat radius; the main influential factors of permeability include throat radius, throat radius uniformity coefficient, aspect ratio and coordination number; the main influential factors of relative permeability include throat radius uniformity coefficient, aspect ratio, wettability, shape of pore throat, and coordination number. It also indicates that wettability and shape of pore throat have a greater impact on irreducible water saturation; aspect ratio and shape of pore throat affect residual oil saturation largely. Additionally, the micro-parameters that influence the ratio of endpoint relative permeability to a great extent are throat radius uniformity coefficient, aspect ratio, and coordination number.

In order to embody the synergetic relationship between reservoir micro-parameters’ variation and macro-parameters’ variation, the influence of various reservoir micro-parameters’ single-factor alteration on reservoir macro-parameters is investigated using network simulation. The relevant results are listed in Table 3 in the form of micro-parameters’ change trend. The symbol “↑” in Table 3 represents that the parameter has an upward trend, meanwhile the symbol “↓” represents that it has a downward trend. Table 3 has embodied the relation between reservoir macro-parameters and the major micro-parameters successfully. For instance, as the scour pore volume multiple increases gradually, throat radius will increase and it will result in the increase of porosity and permeability primarily, which is consistent with the macroscopic trend that porosity and permeability increase gradually during the development of waterflooding.

**Table 3:** Trend of reservoir macro-parameters’ and micro-parameters’ change as waterflooding develops

Influence parameters	*ϕ* (↑)	*K* (↑)	*S*_wc_ (↑)	*S*_or_ (↓)	*K*_rwo_ (↑)
Pore throat radius (↑)	↑	↑			
Homogeneity coefficient of pore throat (↑)		↑			↑
Pore throat ratio (↓)		↑		↓	↑
Wettability (tend to water-wet)			↑		
Shape of pore throat (tend to regular)			↑	↑	
Coordination number (↑)		↑		↓	↑

As can be seen from Table 3, the change trend of macroscopic parameters influenced by microscopic is essentially consistent with that of macro-parameters in water flooding. In other words, the variation rules of macro-parameters in water flooding are actually an integrated reflection of different micro-parameters’ variation. For instance, residual oil saturation decreases with the development of waterflooding, which is mainly attributed to the variability of aspect ratio and coordination number. Although changes of pore throat shape have a negative effect on the reduction of residual oil saturation, the residual oil saturation still declines when it is influenced synthetically.

It is quite difficult to understand the relationship between macro-parameters and micro-parameters in different cases of micro-parameters’ variation. However, for the study of macroscopic flow mechanism and oilfield development rules, if the synergetic time-variability of different macro-parameters is understood, it is enough to establish a comprehensive model which can be used to describe the variability of reservoir macro-parameters. Therefore, the comprehensive model for describing the variability of reservoir macro-parameters is established using massive network simulation in this paper.

Based on the network model as shown in Fig. 1, various network models can be established to carry out microscopic simulation by changing values of the micro-parameters such as throat radius, throat radius uniformity coefficient, aspect ratio, wettability of pore throat, shape factor, and coordination number at random, by which 568 sequences of porosity, permeability, and relative permeability data can be obtained. To analyze the relationship of different data sequences is to establish the comprehensive model, which can describe the influence of micro-parameters’ synthetic variations on reservoir macro-parameters.

As what is investigated is the variability of different macro-parameters, the maximum or minimum of various data sequence is used as the reference value to define different variables. For example, *K*_0_, *ϕ*_0_, *S*_wc0_, *S*_or0_, and *K*_rwo0_ are the initial permeability, porosity, irreducible water saturation, residual oil saturation, and ratio of endpoint relative permeability when neglecting the reservoir parameters’ variations, respectively.

**Fig. 3:**
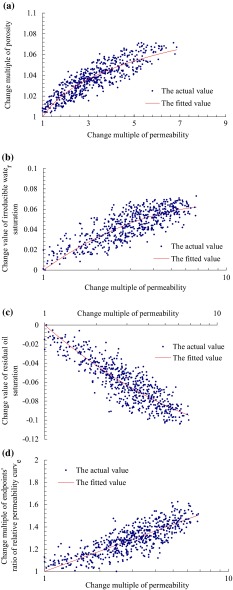
**a** Relationship (change multiple of permeability vs. change multiple of porosity), **b** relationship (change multiple of permeability vs. change value of irreducible water saturation), **c** relationship (change multiple of permeability vs. change value of residual oil saturation), and **d** relationship (change multiple of permeability vs. change multiple of endpoints’ ratio of relative permeability curve)

Using the statistical analysis technique to analyze the results of network simulation (as shown in Fig. 3), the comprehensive models proposed for describing the variability of reservoir macro-parameters are Eqs. (16)–(19).

16 $$R_{\phi } = R_{K}^{0.0325} \left( {R^{2} = 0.7798} \right)$$

17 $$D_{\text{swc}} = - 0.0639(\lg R_{K} )^{2} + 0.1275\lg R_{K} \left( {R^{2} = 0.6201} \right)$$

18 $$D_{\text{sor}} = 0.0525(\lg R_{K} )^{2} - 0.1573\lg R_{K} \left( {R^{2} = 0.7405} \right)$$

19 $$R_{\text{kr}} = - 0.0027(\lg R_{K} )^{2} + 0.6073\lg R_{K} + 1\left( {R^{2} = 0.6375} \right),$$

where *R* is the correlation coefficient of curvilinear regression.

## Results and discussion

**Fig. 4:**
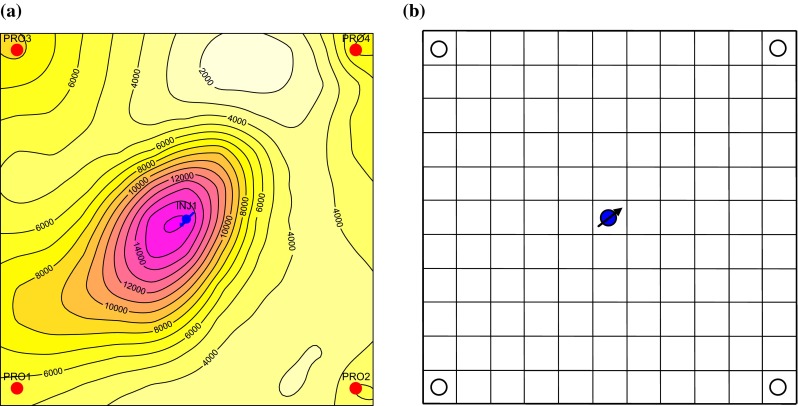
**a** Permeability distribution of the 5th layer in the synthetic five-spot model (10^−3^μm^2^), and **b** schematic diagram of planar grid system in the synthetic five-spot model

In order to discuss the influence of reservoir parameters’ variation on oilfield development rules, a synthetic five-spot model is established with the producer-injector spacing of 212 m. A uniform grid system is adopted in the plane, grid sizes of which are 27.3 m both in *X* direction and *Y* direction. The permeability plane distribution is randomly generated, with its average value of 2 μm^2^ and planar variance coefficient of 0.5. Permeability of different layers is various, but the distribution law is consistent. The permeability plane distribution is shown in Fig. 4a.

There are five oil layers in the longitudinal direction with equal thickness and positive rhythm; the total thickness of the reservoir is 15 m; the variance coefficient of permeability in the longitudinal direction is 0.7. Average permeability from the 1st layer to the 5th layer is 0.62, 1.10, 1.58, 2.29, and 4.41 μm^2^, respectively. The simulation area corresponds to a network system (as shown in Fig. 4b), whose size is 11 × 11 × 5=605. The total injection rate is 100 m^3^/day and the total production rate is 25 m^3^/day for each production well. The injection and production rates of each layer are calculated according to the layer permeability and layer thickness automatically. Results show that:

1. As the scour multiple of injected water increases, permeability and porosity become larger. Generally speaking, porosity and permeability have certain proportional relationship with each other. Due to existence of reservoir heterogeneity, the water volume distributed to the regions or layers with high porosity and permeability is usually large, scour multiple is high, and the variability of permeability and porosity is great. On the contrary, the porosity variation distributed to regions or reservoirs with low porosity and permeability is small, which will lead to the reservoir heterogeneity larger and the development effect worse. Therefore, the oil recovery prediction when considering the variability of permeability or porosity is lower than that of neglecting the variability of reservoir parameters. But compared to permeability, the influence of the variability of porosity on development effect is so small that it can be ignored nearly.
2. As the scour pore volume multiple increases, the development effect becomes better primarily owing to reduction of residual oil saturation. Therefore, the oil recovery prediction when considering the variability of relative permeability is higher than that of neglecting the variability of relative permeability.
3. In condition of neglecting the reservoir parameters’ variation, the recovery prediction of waterflooding is 36.52 % when water cut reaches 98 %. Based on the comprehensive model describing the variability of reservoir macroscopic parameters, the recovery calculated when considering the reservoir parameters’ synergetic variability is 0.6 % higher than that of neglecting the variability of reservoir parameters. Because the variability of relative permeability has a positive effect on development effect, meanwhile the variability of porosity and permeability has a negative impact on development effect, the influence of them on oil recovery is compensated with each other to a certain extent.

The influence of reservoir parameters’ variation on development effect either amplifies or reduces in different reservoir conditions, so there exists a problem that when the variability of reservoir parameters should be taken into account. The influence of reservoir parameters’ variation on development effect will be investigated by altering reservoir heterogeneity and oil viscosity.

### Influence of reservoir heterogeneity

The influence of reservoir parameters’ variation on development effect under different variance coefficients is discussed in positive rhythm formation and reverse rhythm formation, respectively. Positive rhythm formation is defined as the formation in which the permeability increases with reservoir depth. Reverse rhythm formation is defined as the formation in which the permeability decreases as reservoir depth increases.

#### Positive rhythm formation

In the positive rhythm formation, when considering or neglecting the reservoir parameters’ variation, the production performance is simulated in different cases of variance coefficient, which is, respectively, 0, 0.3, 0.5, 0.7, and 0.9.

**Fig. 5:**
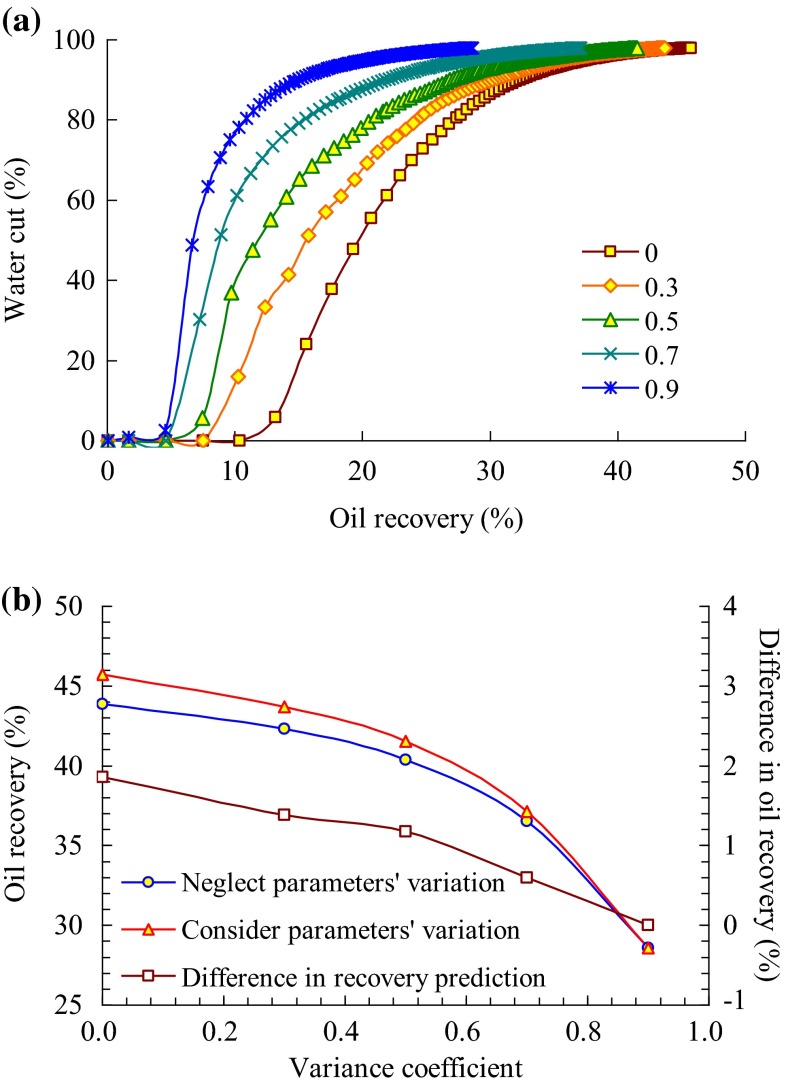
**a** Relationship between water cut and oil recovery under different variance coefficients of positive rhythm formation, and **b** influence of positive rhythm formation parameters’ variation on oil recovery

Relationship between water cut and oil recovery under different variance coefficients when considering reservoir parameters’ variation is shown in Fig. 5a. As the variance coefficient increases, the reservoir heterogeneities enhance, the development effect declines, and the recovery prediction decreases. Figure 5b and Table 4 show the variability of the influence degree of reservoir parameters’ alteration on development effect under different variance coefficients. Results show that as the variance coefficient increases, the reservoir heterogeneities enhance, and the distribution of injected water becomes more disproportional. When considering the variability of reservoir parameters, the distribution of permeability and porosity tends to be more heterogeneous, namely the negative impact of the variability of permeability and porosity on development effect becomes larger. Comparatively speaking, the positive impact of relative permeability variation on development effect tends to be smaller. Therefore, as a whole, with the increase of the variance coefficient, the difference in recovery prediction between considering and neglecting the variability of reservoir parameters tends to be increasingly smaller. When the variance coefficient is 0.9, the difference varies to zero, which means that the negative impact of the variability of permeability and porosity on development effect just compensates the positive impact of relative permeability variation on development effect.

**Table 4:** Influence of positive rhythm formation parameters’ synthetic variation on oil recovery under different variance coefficients

Variance coefficient	Oil recovery prediction (%)		Difference in recovery prediction (%)
	Neglect parameters’ variation	Consider parameters’ variation	
0	43.86	45.72	1.86
0.3	42.32	43.70	1.38
0.5	40.35	41.53	1.18
0.7	36.52	37.12	0.60
0.9	28.60	28.60	0.00

#### Reverse rhythm formation

In the reverse rhythm formation, when considering or neglecting the reservoir parameters’ variation, the production performance is simulated in different cases of variance coefficient, which is, respectively, 0, 0.3, 0.5, 0.7, and 0.9.

**Fig. 6:**
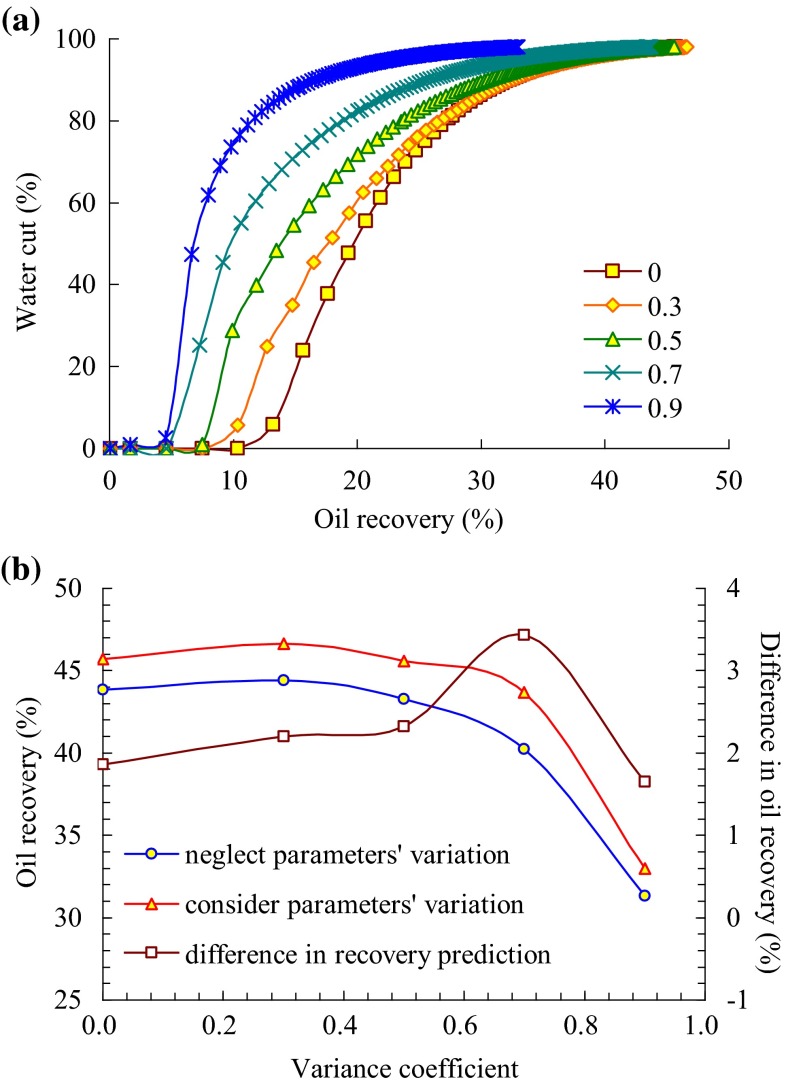
**a** Relationship between water cut and oil recovery under different variance coefficients of reverse rhythm formation, and **b** influence of reverse rhythm formation parameters’ variation on oil recovery

Relationship between water cut and oil recovery under different variance coefficients when considering reservoir parameters’ variation is shown in Fig. 6a. As the variance coefficient increases, the recovery prediction increases first and decreases afterwards, and it will reach to the peak when the variance coefficient is 0.3. This is mainly because the permeability distribution of reverse rhythm formation is the top high and the bottom low, and thus, the injected water tends to flow at the top, whereas the influence of gravity is just contrary. When the variance coefficient is 0.3, the distribution of injected water in different layers which is influenced simultaneously by both factors is relatively homogeneous, and the development effect is the best.

Figure 6b and Table 5 show the variability of the influence degree of reservoir parameters’ alteration on development effect under different variance coefficients. It can be seen that as for the reverse rhythm formation, owing to the synthetic influence of gravity, it is not the higher the initial permeability, the larger the water volume distributed when the variance coefficient takes value at some interval. If it is like this, when considering reservoir parameters’ variation, the distribution of parameters such as permeability and porosity may develop toward the more homogeneous, namely the negative influence of the variability of permeability and porosity on development effect may weaken, and even varies to a positive influence. Therefore, on the whole, with the increase of the variance coefficient, the difference between the recovery prediction when considering the variability of reservoir parameters and that of neglecting the variability of reservoir parameters has a peak. When the variance coefficient is 0.7, the difference in recovery prediction is the largest.

**Table 5:** Influence of reverse rhythm formation parameters’ synthetic variation on oil recovery under different variance coefficients

Variance coefficient	Oil recovery prediction (%)		Difference in recovery prediction (%)
	Neglect parameters’ variation	Consider parameters’ variation	
0	43.86	45.72	1.86
0.3	44.42	46.62	2.20
0.5	43.28	45.60	2.32
0.7	40.24	43.67	3.43
0.9	31.32	32.97	1.65

### Influence of oil viscosity

When considering or neglecting the reservoir parameters’ variation, the production performance is simulated in different cases of oil viscosity, which is, respectively, 5, 20, 40, 150, and 300 mPa s.

**Fig. 7:**
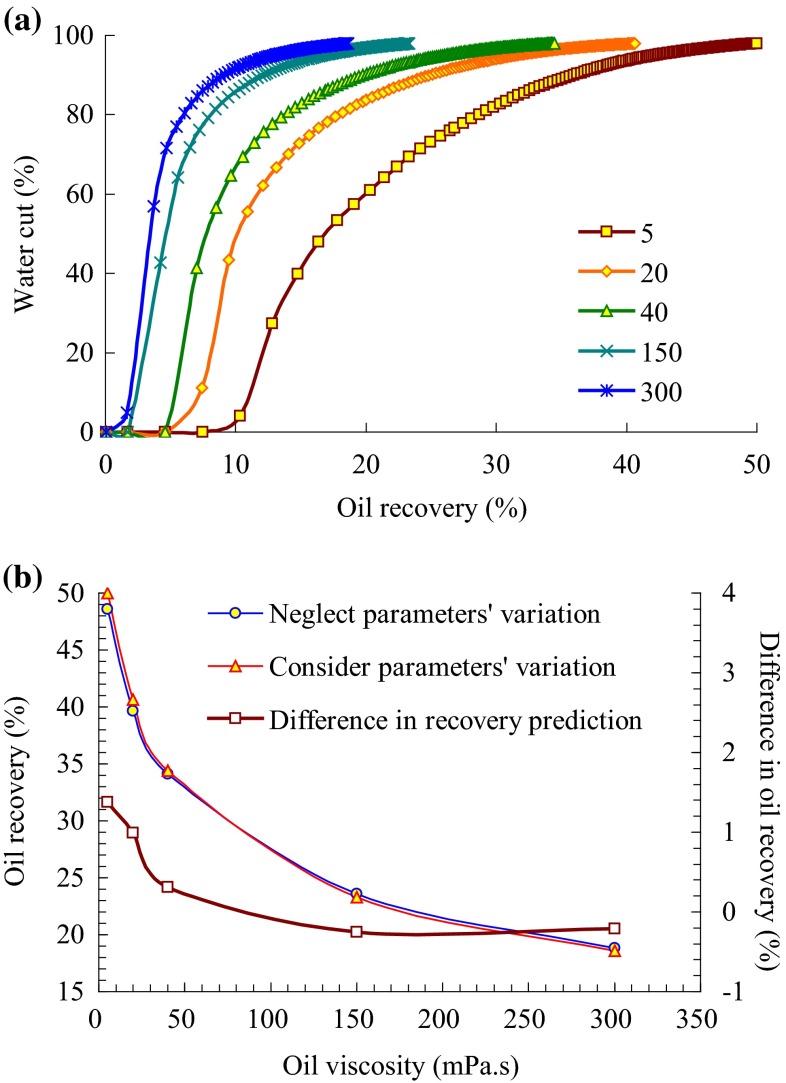
**a** Relationship between water cut and oil recovery under different oil viscosity, and **b** influence of reservoir parameters’ synthetic variation on oil recovery under different oil viscosity

Relationship between water cut and oil recovery under different oil viscosity when considering the reservoir parameters’ variation is shown in Fig. 7a. As oil viscosity increases, water–oil mobility ratio becomes larger, the development effect weakens, and the recovery prediction declines.

Figure 7b and Table 6 show the variability of the influence degree of reservoir parameters’ alteration on development effect under different oil viscosity. It indicates that as oil viscosity increases, frontal movement of injected water becomes worse. When considering the reservoir parameters’ variation, especially the variability of permeability, the negative impact of oil viscosity on development effect is aggravated. Therefore, with the increase of oil viscosity, difference in recovery prediction when considering or neglecting the reservoir parameters’ variation is smaller. However, when oil viscosity reaches to 150 mPa s, the change trend is weakened. Meanwhile, difference in recovery prediction has changed to a negative value, which denotes that the negative impact of the variability of permeability and porosity on development effect is greater than the positive impact of the variability of relative permeability on development effect.

**Table 6:** Influence of formation parameters’ synthetic variation on oil recovery under different oil viscosity

Oil viscosity (mPa s)	Oil recovery prediction (%)		Difference in recovery prediction (%)
	Neglect parameters’ variation	Consider parameters’ variation	
5	48.6	49.98	1.38
20	39.66	40.65	0.99
40	34.13	34.44	0.31
150	23.57	23.32	−0.25
300	18.82	18.61	−0.21

## Conclusions

1. Based on microscopic network simulation and reservoir numerical simulation, a simulation method is proposed, which can describe the influence of reservoir parameters’ synergetic time-variability on oil development rules in microscopic and macroscopic scales. The study on reservoir parameters’ time-variability provides an effective simulation technique to establish the internal relationship of reservoir micro-parameters, macro-parameters, and oilfield development rules.
2. The macroscopic parameters such as porosity, absolute permeability, and relative permeability are used as constraint conditions to generate a microscopic network model of oil–water two-phase flow, which can effectively describe the influence of reservoir micro-parameters’ variation on macro-parameters. Based on massive network simulation, a comprehensive model is established to describe the time variability of reservoir macro-parameters, which shows the synergetic relationship between the variability of porosity and relative permeability and that of permeability.
3. Taking into consideration the time variability of porosity, permeability, and relative permeability in waterflooding, the influence of reservoir parameters’ synergetic variation on development rules is discussed based on the improved reservoir numerical simulator of oil–water two-phase flow.
4. The effect of reservoir parameters’ synthetic variation on oilfield development rules is the consolidated reflection of each single factor’s effect on oilfield development rules. These single factors include porosity, permeability, and relative permeability. In positive rhythm formations, as the variance coefficient increases, the synthetic influence of reservoir parameters on oil development rules weakens gradually. However, in reverse rhythm formations, the synthetic influence on oil development rules has a peak when the variance coefficient is different. As oil viscosity increases, the negative impact of permeability variation on production performance can be further aggravated, and the synthetic influence is that the difference in recovery prediction when considering or neglecting the variability of reservoir parameters weakens.
